# Sex and estrogens alter the action of glucagon-like peptide-1 on reward

**DOI:** 10.1186/s13293-016-0059-9

**Published:** 2016-01-16

**Authors:** Jennifer E. Richard, Rozita H. Anderberg, Lorena López-Ferreras, Kajsa Olandersson, Karolina P. Skibicka

**Affiliations:** Department of Physiology/Metabolic Physiology, Institute of Neuroscience and Physiology, The Sahlgrenska Academy at the University of Gothenburg, Medicinaregatan 11, PO Box 434, SE-405 30 Gothenburg, Sweden

**Keywords:** Glucagon-like peptide-1, GLP-1, Exendin-4, Reward, Estrogens, Sex, Obesity

## Abstract

**Background:**

Feeding behavior is regulated through an intricate array of anorexic and orexigenic hormones acting on the central nervous system (CNS). Some of these hormones may have differential effects in males and females, effects potentially attributed to actions of gonadal steroids, especially estrogens. Central stimulation of the glucagon-like peptide-1 (GLP-1) receptors reduces feeding and food-reward behavior by acting on CNS regions important for the anorexic actions of estrogens. Thus, we propose that the action of GLP-1 on food intake and reward may differ between sexes.

**Methods:**

Male and female rats were centrally injected with the GLP-1 analog exendin-4 (Ex4) in a non-deprived or food-restricted state; reward behavior was measured in a progressive ratio operant conditioning task. Intake of chow and palatable food were also measured. To determine if sex differences in the actions of Ex4 are due to interactions with estrogens, Ex4 treatment was preceded by treatment with a nonselective estrogen receptor-α (ERα) and ERβ or ERα-selective antagonist.

**Results:**

Central injection of Ex4 revealed increased reward behavior suppression in females, compared to males, in the operant conditioning task. This increase was present in both non-deprived and food-restricted animals with larger differences in the fed state. Intake of chow and palatable food, after Ex4, were similar in males and females. Food reward, but not food intake, effect of Ex4 was attenuated by pretreatment with ER antagonist in both sexes, suggesting that estrogens may modulate effects of Ex4 in both sexes. Furthermore, central pretreatment with ERα-selective antagonist was sufficient to attenuate effects of Ex4 on reward.

**Conclusions:**

Collectively, these data reveal that females display much higher sensitivity to the food reward impact of central GLP-1 receptor activation. Surprisingly, they also demonstrate that central ERα signaling is necessary for the actions of GLP-1 on food-reward behavior in both sexes.

## Background

Sex is a basic biological variable that influences physiology and disease. Despite the overrepresentation of women in diseases resulting from disordered eating [1, 2], few preclinical studies have a clear focus to explore the neurobiology and physiology of food intake regulation in females. This sex gap is symptomatic of what is seen in preclinical medical research overall. According to a recent review of preclinical publications [3], nearly two out of three papers did not even report the sex of the animals used in the study. Nevertheless, physiology and pathophysiology of feeding behavior differ in male and female animals or humans. Recent data suggest that the female brain responds differently to different food intake regulating signals [4–6].

Glucagon-like peptide-1 (GLP-1) and its receptors have emerged as a successful therapeutic target for treatment of type-2 diabetes [7, 8]. The usage of GLP-1-based therapy is increasing at a fast pace; however, some aspects of GLP-1 function, especially pertaining to its role in the central nervous system (CNS), remain unexplored. Considering the increasing amount of patients receiving this treatment [9, 10], there is a certain urgency to develop a better understanding of the action of GLP-1 and its analogs on the CNS. Increasingly, more evidence is pointing to the CNS as a major target for GLP-1 [11]. In fact, derived from preproglucagon, GLP-1 is produced not only in the periphery (intestine, pancreas) but also in the CNS, primarily by neurons located in the nucleus of the solitary tract (NTS). Central injections of GLP-1 receptor (GLP-1R) agonists potently decrease food intake; this anorexic action has been ascribed to the hypothalamic and brainstem GLP-1R expressing targets [11–15]; the same CNS regions are implicated for the anorexic action of estrogens [16–19], providing neuroanatomical grounds for a potential interaction between GLP-1R activation, sex, and estrogens proposed here.

Food intake is regulated not only from brain regions traditionally recognized for their role in homeostatically driven feeding, like the hypothalamus and the hindbrain, but also from extra-homeostatic areas that control rewarding aspects of eating [20, 21]. Food reward is an important component in the development of overeating and obesity [22] and can be divided in two components, “liking” and “wanting,” as previously described by Berridge et al. [23, 24]. Wanting is associated with incentive salience and motivation for a certain type of food and is often coupled to stimuli, which can trigger a desire to acquire or work for the rewarding component. Liking on the other hand is more commonly associated with palatability. Though these two systems are closely linked, they also have the ability to act independently of each other, and via separate neural pathways, to modulate reward [23]. The wanting component of food reward is closely linked to the mesolimbic neurocircuitry, especially the ventral tegmental area (VTA) and its dopaminergic projections to the nucleus accumbens (NAc) [25]. GLP-1Rs and GLP-1-carrying fibers can also be found in these areas of the brain, involved in motivated behavior and addiction [26]. GLP-1 can change reward behavior in males [27–31]. However, little is known about GLP-1-driven food-reward control in females. Here, we determined whether there are sex differences in the sensitivity of food reward, more specifically food motivation and consumption of palatable food, and chow intake behavior to central GLP-1R activation.

## Methods

### Animals

Female and male Sprague-Dawley rats (160–200 g at arrival, and mean body weights of 260 and 480 g for females and males, respectively, during testing, Charles River, Germany) were housed in a 12-h light/dark cycle, in individual cages with ad libitum access to chow and water, unless otherwise specified. Female rats presented with normal 4- to 5-day estrous cycles throughout the experimental testing. However, this study was not designed to analyze any potential impact of cycling. All testing was conducted during the light cycle. All studies were carried out with ethical permissions from the Animal Welfare Committee of the University of Gothenburg, in accordance with legal requirements of the European Community (Decree 86/609/EEC). All efforts were made to minimize suffering.

### Drugs

Exendin-4, angiotensin II, ICI 182, 780 [32], and MPP dihydrochloride (MPPd) were purchased from Tocris (Bristol, UK). Ex4 and angiotensin II were dissolved in artificial cerebral spinal fluid (aCSF, vehicle for central injection), and ICI and MPPd were dissolved in aCSF with 10 and 20 % DMSO, respectively. All drugs were stored as aliquots at −20 °C.

### Operant conditioning

The progressive ratio operant conditioning schedule is a procedure used to analyze motivated behavior (reward wanting, often compared to the human experience of craving) and measures the amount of work or effort that a subject is willing to put in to obtain a reward, in this case rewarding food in the form of sucrose, and it therefore mainly measures the wanting component of food reward. Operant conditioning training was conducted in rat conditioning chambers (Med-Associates, Georgia, VT, USA) as described previously [27, 33] in ad libitum fed rats. Rats were trained to press a lever for a 45-mg sucrose pellet. Training was conducted in four stages: rats were first trained on the fixed ratio 1 (FR1) schedule in 30-min sessions (single press on the active lever resulted in the delivery of one sucrose pellet), followed by FR3 and FR5 (3 and 5 presses per pellet, respectively), where a minimum of 30 responses per session on the active lever was required for advancement to the next schedule, concluding with progressive ratio conditioning until stable responding was achieved. Each progressive ratio session lasted for 1 h. Responding was considered stable when the number of pellets earned per session did not differ more than 15 % between three consecutive sessions. All operant response testing was performed after the responses stabilized. All drug injections and testing were performed during the light cycle, starting 2 h after lights-on as specified below, and testing commenced 20 min after drug injection. Injections were done in a counterbalanced, Latin square design, with at least 48 h separating each injection condition.

### Brain cannulation

Due to variations in body weight between male and female subjects, in addition to differential fat pad location and size [34], along with the fact that Ex4 can cross the blood-brain barrier [35], direct brain administration was chosen for all substances tested in order to avoid the potential confounding effects from these variations. As a result, our experiments are not relevant to effects solely mediated by peripheral GLP-1R. Rats were implanted with a guide cannula targeting the lateral ventricle [36] (26 gauge; Plastics One, Roanoke, VA): ±1.6 from the midline, 0.9 mm posterior to bregma, and 2.0 mm ventral to skull, with an injector aimed 4.0 mm ventral to the skull under ketamine anesthesia. The cannulas were attached to the skull with dental acrylic and jeweler’s screws and closed with an obturator as described previously [37]. Placement was verified with the angiotensin II drinking test. Angiotensin II was injected at a dose of 20 ng in 2 μL of aCSF, and water intake was measured throughout the following 30 min. Rats who consumed a minimum of 5 mL of water within the measured time period were considered to have correctly positioned cannulas.

### Effects of central Ex4 in *non-deprived* males and females on food-motivated behavior and food intake

Male and female rats were injected with Ex4 (0.1 or 0.3 μg/μL) or vehicle into the left ventricle (LV). Doses were previously shown to reduce food motivation in food-restricted male rats [27]. Twenty minutes after injection, the rats were placed in the operant conditioning chambers for a 1-h testing period. Testing was performed in a non-deprived state 2 h following the dark cycle period where rats had ad libitum access to chow. After testing, the rats were returned to their home cages and given access to palatable food—peanut butter. After 1 h, the amount of consumed peanut butter was measured and the rats received normal chow for the remainder of the 24-h period.

### Effect of central Ex4 in overnight *food-restricted* males and females on food-motivated behavior and food intake

Rats were food-restricted overnight to approximately 50 % of their normal intake. On the following day, rats of each sex were injected with Ex4 (0.3 μg/μL) or vehicle and the operant conditioning test was performed 20 min after injection for 60 min. Peanut butter intake and chow intake were measured as described above.

### Effects of central estrogen blockade on male and female food-motivated behavior after Ex4 treatment

Food-restricted male and female rats received injections of the estrogen receptor (ER) antagonist ICI (10 μg/μL) [38] or vehicle into the left ventricle (LV) prior to injection of Ex4 (0.3 μg/μL) or vehicle in this area. Food restriction and the higher Ex4 dose were chosen in order to induce reliable reward behavior reduction in both sexes. Operant conditioning, intake of peanut butter, and chow consumption were measured as described above.

### Effects of central administration of the ERα-antagonist on male and female food-motivated behavior after Ex4 treatment

Overnight food-restricted rats were injected with 1.4 μg/μL [39] of the estrogen receptor-α (ERα) (Esr1)—antagonist MPPd or vehicle, accompanied by injection of 0.3 μg/μL Ex4 or vehicle, into the LV. Operant conditioning, intake of peanut butter, and chow consumption were measured as described above.

### Statistical analysis

All the data are presented as mean ± standard error of the mean (SEM). Statistical significance was analyzed using Student’s *t* test or one- and two-way ANOVA, when appropriate, with Sidak’s or Holm-Sidak’s post hoc tests (GraphPad Software, Inc., San Diego, CA). *p* values lower than 0.05 were considered statistically significant.

## Results

### Effect of central Ex4 treatment on reward and food intake in *non-deprived* males and females

**Fig. 1:**
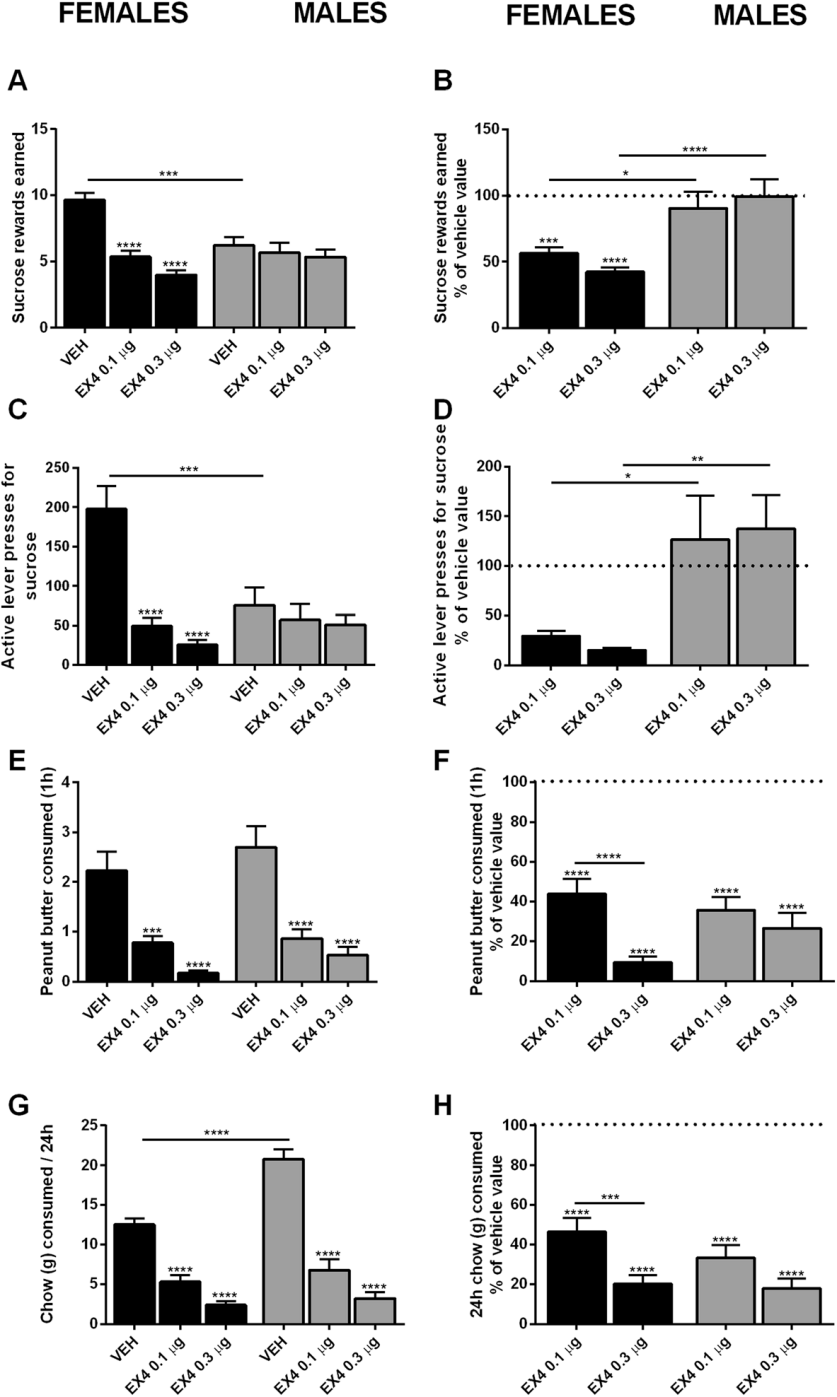
In non-deprived animals, central GLP-1R stimulation specifically decreases food-motivated behavior in females but not males. Ex4 injection into the LV significantly reduced sucrose rewards earned (**a**), and active lever presses for reward (**c**) in female but not male subjects. Sucrose rewards (**b**) and active lever presses (**d**) were also significantly lower in females compared to males after Ex4 treatment, at both doses, analyzed as percent relative to vehicle value. Central GLP-1R agonist treatment significantly reduced peanut butter consumption in both males and females (**e**), with no significant differences between the two sexes (**f**). Chow consumption was reduced in males and females after Ex4 treatment (**g**); no significant differences were present between sexes at either dose of the agonist (**h**). Chow and peanut butter consumption are displayed in grams. Data are expressed as mean ± SEM. *n* = 20 (females) and 18 (males) per treatment group. *p < 0.05, **p < 0.01, ***p < 0.001, ****p < 0.0001

To investigate potential differences in the effects of central Ex4 treatment between males and females, rats of both sexes were tested using a progressive ratio reinforcement schedule after administration of Ex4 (0.01 or 0.03 μg/μL) or vehicle. Two-way repeated measures ANOVA revealed a significant reduction in sucrose rewards earned, and lever presses for sucrose, at both doses of Ex4 in female, but not male, rats (Holm-Sidak’s multiple comparisons test, *p* < 0.0001; Fig. 1a, c). The main effect of drug treatment was significant for both rewards earned and number of lever presses for rewards (*F*_(2, 108)_ = 19.25, *p* < 0.0001; *F*_(2, 108)_ = 15.91, *p* < 0.0001). The main effect of sex on rewards earned and lever presses was not significant (*F*_(1, 108)_ = 1.790, *p* > 0.05; *F*_(1, 108)_ = 3.717, *p* > 0.05, respectively). Importantly, there was a significant interaction between these two factors (*F*_(2, 108)_ = 10.46, *p* < 0.0001; *F*_(2, 108)_ = 9.195, *p* < 0.001). Data displayed as sucrose rewards earned and active lever presses in percent of vehicle value revealed significant differences for both parameters at both doses of Ex4 between males and females (*p* < 0.05, Fig. 1b; *p* < 0.05, Fig. 1d). Two-way ANOVA also revealed a significant effect of sex (*F*_(1, 108)_ = 25.56, *p* < 0.0001; *F*_(1, 108)_ = 17.02, *p* < 0.0001) and a significant interaction between sex and treatment (*F*_(2,108)_ = 7.610, *p* < 0.001; *F*_(2,108)_ = 4.424, *p* < 0.05).

Central GLP-1R stimulation led to a significant decrease in peanut butter consumption compared to controls in both sexes (two-way ANOVA, Holm-Sidak’s multiple comparisons test, *p* < 0.001 and *p* < 0.0001 for females for the low and high dose of Ex4, respectively, and *p* < 0.0001 for males for both doses of Ex4; Fig. 1e). Two-way repeated measures ANOVA indicated that the main effect of sex on peanut butter consumption was not significant (*F*_(1, 108)_ = 2.088, *p* > 0.1). The effect of drug treatment, however, was significant (*F*_(2, 108)_ = 36.38, *p* < 0.0001) though there was not a significant interaction between these two factors (*F*_(2, 108)_ = 0.2951, *p* > 0.1). Data expressed as percent of vehicle value revealed a significant effect of drug treatment (*F*_(2, 108)_ = 130.1, *p* < 0.0001), but not the effect of sex (*F*_(1, 108)_ = 0.4864, *p >* 0.1; Fig. 1f) or interaction between these parameters (*F*_(2, 108)_ = 3.026, *p* > 0.05).

To investigate if 1 h peanut butter consumption is influenced by the experimental setup (the timing of the test relative to injections or the fact that it is preceded by the operant task), an additional group of males received Ex4 injections (0.3 μg/μL) or vehicle, in a non-deprived state, and peanut butter consumption was measured 20 min after injection for 60 min (the same time point as operant conditioning testing was conducted). One-hour peanut butter consumption was significantly reduced in Ex4-treated male subjects compared to controls (Student’s *t* test, *p* < 0.05, data not shown) at this time point. The size of the effect was comparable to the effect obtained when testing was conducted after PR, indicating that 1 h intake after PR testing was not affected by the experimental design.

In addition to peanut butter, Ex4 treatment led to a significant dose-dependent reduction in chow intake after 24 h, in both male and female rats (two-way ANOVA, Holm-Sidak’s multiple comparisons test, *p* < 0.0001 for both doses of Ex4, in males and females; Fig. 1g). The main effect of drug treatment was significant (two-way ANOVA, *F*_(2, 108)_ = 118.5, *p <* 0.0001). Two-way repeated measures ANOVA also revealed a significant difference between males and females in both the main effect of sex (*F*_(1, 108)_ = 21.01, *p* < 0.0001) as well as interaction between sex and drug treatment (*F*_(2, 108)_ = 9.590, *p* < 0.001). Comparison of chow consumption relative to vehicle revealed a significant effect of treatment (*F*_(2, 108)_ = 149.5, *p* < 0.0001; Fig. 1h), but not sex (*F*_(1, 108)_ = 1.698, *p* > 0.1), and there was no significant interaction between these two factors (*F*_(2, 108)_ = 1.051, *p* > 0.1).

### Effect of central Ex4 treatment on reward and food intake in *food-restricted* males and females

**Fig. 2:**
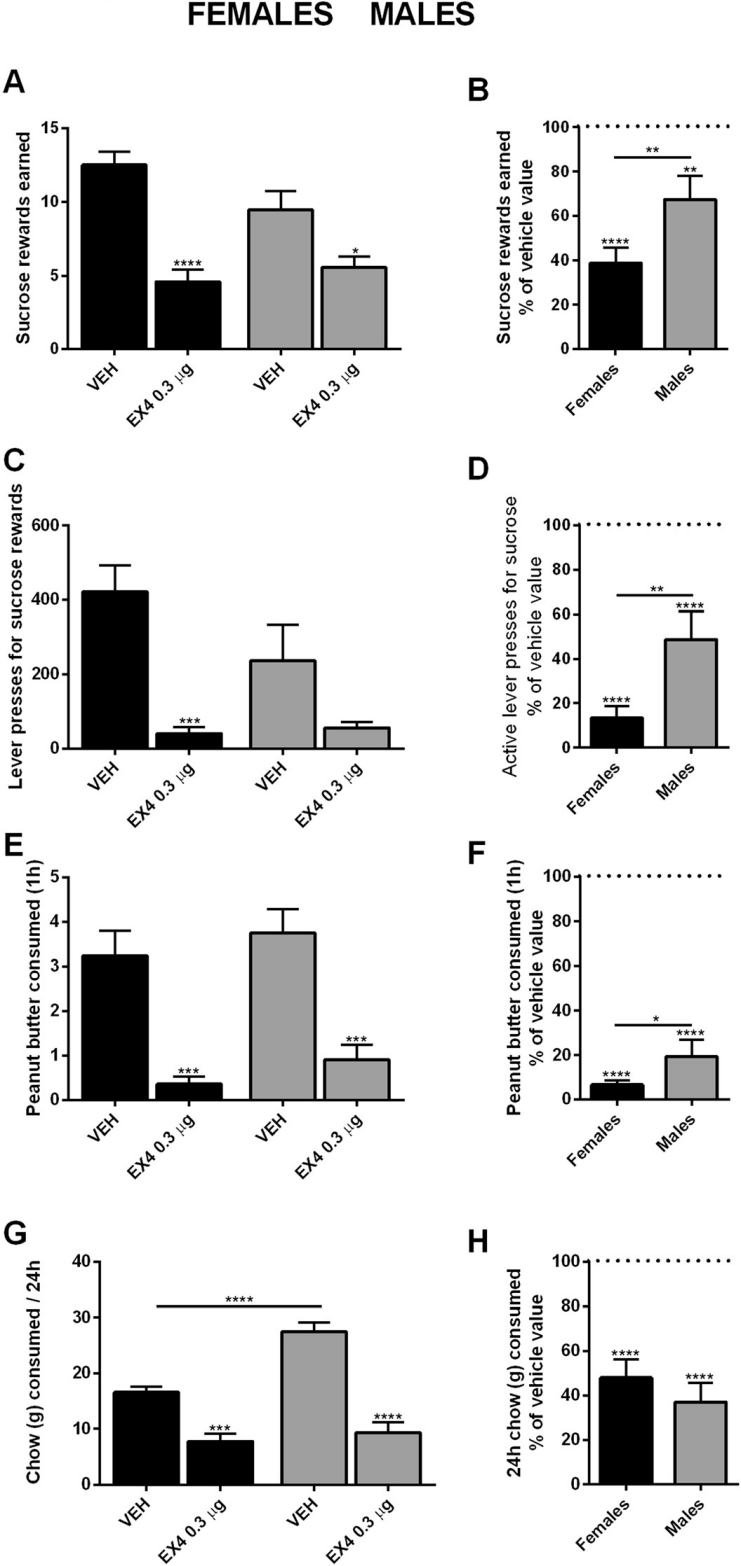
Ex4 treatment in overnight food-restricted rats leads to a larger reduction in food-motivated behavior in females than males. After overnight food restriction, central Ex4 treatment reduced sucrose rewards earned in both males and females (**a**), an effect that was larger in females (**b**). Active lever presses were significantly reduced by Ex4 treatment in female subjects; a trend for reduction was present in male subjects but it did not reach statistical significance (**c**). Lever presses after Ex4 treatment in males and females analyzed as percent of vehicle treatment also revealed a significantly larger reduction in female subjects (**d**). Peanut butter consumption was significantly reduced in both sexes (**e**) with a slightly larger response to Ex4 in females (**f**). Twenty-four hour chow consumption was reduced in both males and females after GLP-1R agonist injection (**g**); no significant differences were found in chow intake between the two sexes (**h**). Chow and peanut butter consumption are displayed in grams. Data are expressed as mean ± SEM. *n* = 10 (females) and 9 (males) per treatment group. *p < 0.05, **p < 0.01, ***p < 0.001, ****p < 0.0001

Due to the absence of effect of Ex4 on food-motivated behavior in non-food deprived males, an effect which has previously been established for fasted males, additional experiments in a food-restricted state were necessary to validate the experimental setup and to investigate potential differences in the action of Ex4 between sexes in different hunger states. Operant conditioning testing as well as food intake measurements were therefore conducted in overnight food restricted animals using only the higher concentration of Ex4. Under these conditions, Ex4 significantly decreased the amount of sucrose rewards earned in both males and females (Holm-Sidak’s multiple comparisons test, *p* < 0.0001 and *p* < 0.05 for females and males, respectively; Fig. 2a). While the main effect of drug treatment was significant (two-way ANOVA, *F*_(1, 34)_ = 38.41, *p* < 0.0001), the main effect of sex was not (*F*_(1, 34)_ = 1.219, *p* > 0.1). Importantly, there was also a significant interaction between these two factors (*F*_(1, 34)_ = 4.447, *p* < 0.05). Though significant in both groups, females had a larger reduction in rewards earned than males (sucrose rewards earned as percent of vehicle value, *p <* 0.01; Fig. 2b). In the restricted condition, lever pressing for sucrose rewards reached significance in the female group after analysis using two-way ANOVA analysis (*p <* 0.001; Fig. 2c). Though the effect of treatment was significant (*F*_(1, 34)_ = 21.66, *p* < 0.0001), there was no significant effect of sex (*F*_(1, 34)_ = 1.943, *p* > 0.1) or interaction of sex and drug treatment (*F*_(1, 34)_ = 2.725, *p* > 0.1). Additionally, a significant difference was present between active lever press counts for restricted males and females (presses in percent of vehicle, *p <* 0.01; Fig. 2d).

In restricted animals, Ex4 treatment led to a significant reduction in 1 h peanut butter consumption in both sexes (two-way ANOVA, Holm-Sidak’s multiple comparisons test, *p* < 0.001 for males and females; Fig. 2e). The main effect of drug treatment was significant (two-way ANOVA, *F*_(1, 34)_ = 44.26, *p <* 0.0001). The main effect of sex was non-significant (*F*_(1, 34)_ = 1.545, *p* > 0.1). There was no significant interaction between these two factors (*F*_(1, 34)_ = 0.00183, *p* > 0.1). There was, however, a difference in peanut butter consumption between male and female Ex4-treated groups when analyzed as percent peanut butter consumed relative to the amount consumed after vehicle injection (*p* < 0.05; Fig. 2f).

Ex4 treatment also led to a significant reduction in 24 h chow consumption, compared to controls, in both restricted males and females (two-way ANOVA, Holm-Sidak’s multiple comparisons test, *p* < 0.001 and *p* < 0.0001 for females and males, respectively; Fig. 2g). Two-way ANOVA revealed significance in all measures, including the main effect of sex (*F*_(1, 34)_ = 18.46, *p* < 0.001), the main effect of drug treatment (F_(1, 34)_ = 85.50, *p* < 0.0001), and the interaction between these two measures (*F*_(1, 34)_ = 10.14, *p* < 0.01). However, no differences were found between Ex4-treated males and females when displayed as percent chow consumed compared to vehicle (Fig. 2h).

### Effect of estrogen blockade on the actions of Ex4 on reward in food-restricted rats

**Fig. 3:**
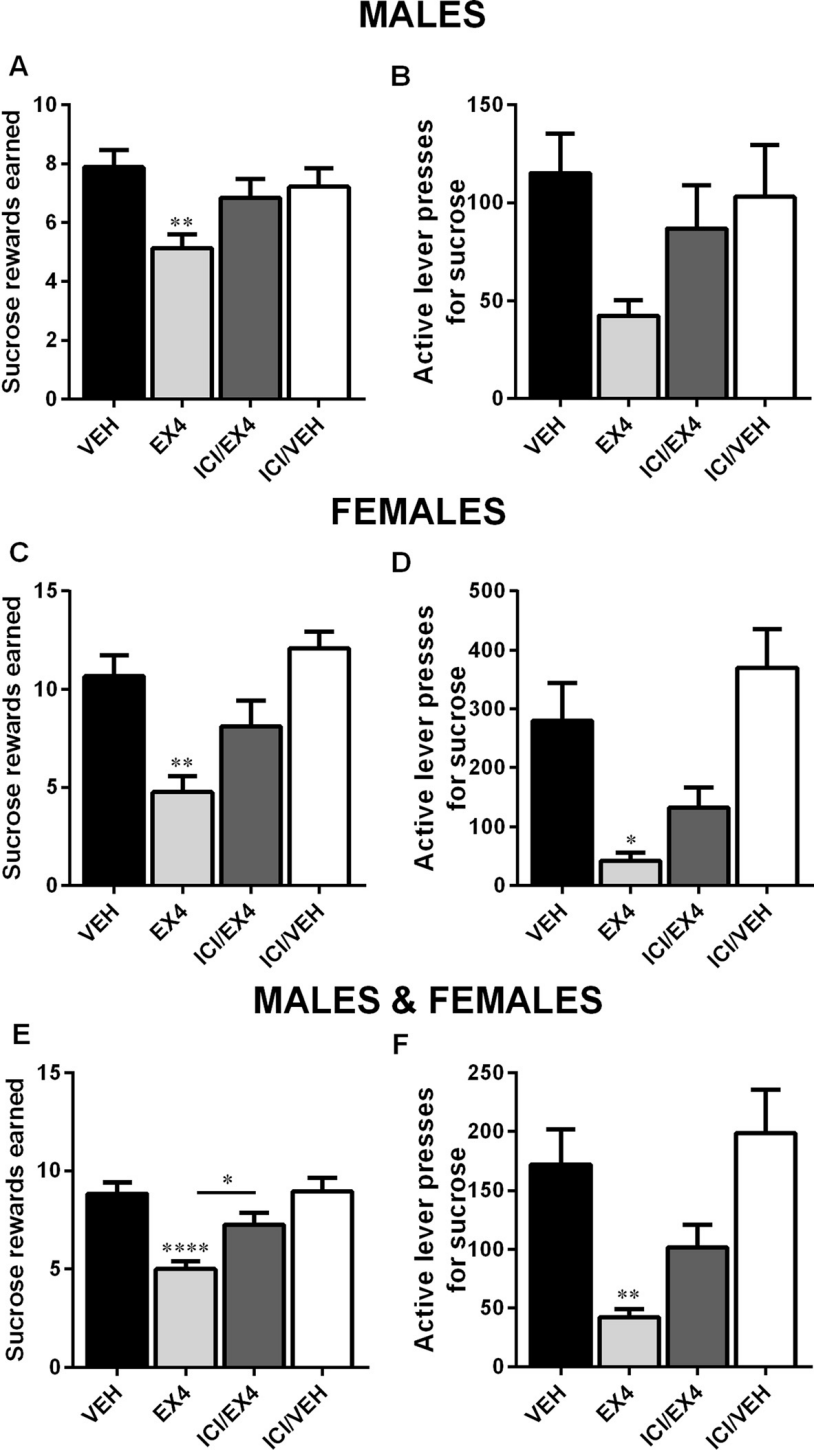
Central estrogen blockade attenuates the effects of Ex4 on food-motivated behavior. Concurrent administration of the selective estrogen antagonist ICI and Ex4 attenuated the reduction in sucrose rewards earned in females (**c**) and males (**a**) compared to Ex4 treatment alone, although this effect did not reach significance. Reduction of active lever presses for sucrose was also attenuated in both sexes after ICI treatment (**b**, **d**). The number of sucrose rewards earned for males and females combined was reduced after central Ex4 injection (**e**); this effect was attenuated after ICI treatment. Combined results for males and females also demonstrate a significant reduction in lever presses for sucrose after central GLP-1R stimulation; attenuation by the ICI treatment did not reach statistical significance (**f**). Data are expressed as mean ± SEM. *n* = 10 (females) and 18 (males) per treatment group. *p < 0.05, **p < 0.01, ****p < 0.0001

To explore the impact of estrogens on the actions of Ex4, the estrogen antagonist ICI was administered centrally along with Ex4 into fasted male and female rats. Two-way repeated measures ANOVA revealed a significant difference in the main effect of sex (*F*_(3, 98)_ = 14.71, *p* < 0.001) and effect of drug treatment (*F*_(3, 98)_ = 14.43, *p* < 0.0001). A significant interaction between the two factors was also detected (*F*_(3, 98)_ = 4.014, *p* < 0.01). Pretreatment with ICI attenuated the Ex4-induced food reward suppression in female rats. After the combined treatment, the amount of sucrose rewards earned was therefore no longer statistically significant compared to controls (Fig. 3c). Interestingly, similar effects of the combined treatment on sucrose rewards earned were also present in males (Fig. 3a). For lever presses, two-way ANOVA indicated a significant effect of sex (*F*_(1, 98)_ = 25.87, *p* < 0.0001) and effect of drug treatment (*F*_(3, 98)_ = 13.87, *p* < 0.0001). Importantly, there was also a significant interaction between the two factors (*F*_(3, 98)_ = 6.792, *p* < 0.001). ICI treatment in females led to an attenuation of the reduction of active lever presses for sucrose after central GLP-1R stimulation (Fig. 3d). In males, the reduced amount of active lever presses for sucrose did not reach statistical significance after Ex4 treatment, although interestingly, combination of Ex4 with the antagonist treatment did lead to an increase in the amount of lever presses compared to Ex4 treatment alone (Fig. 3b). When results of males and females are combined due to similar trend in response pattern, a significant difference in rewards earned (one-way ANOVA, *F*_(3, 102)_ = 9.633, *p* < 0.05; Fig. 3e), but not active lever presses for sucrose (Fig. 3f), between Ex4- and Ex4/ICI-treated rats is detected. ICI treatment alone did not have any significant effects on reward-related behavior in this experiment. One male rat and three female rats did not finish the study due to dislodged or blocked cannula.

**Fig. 4:**
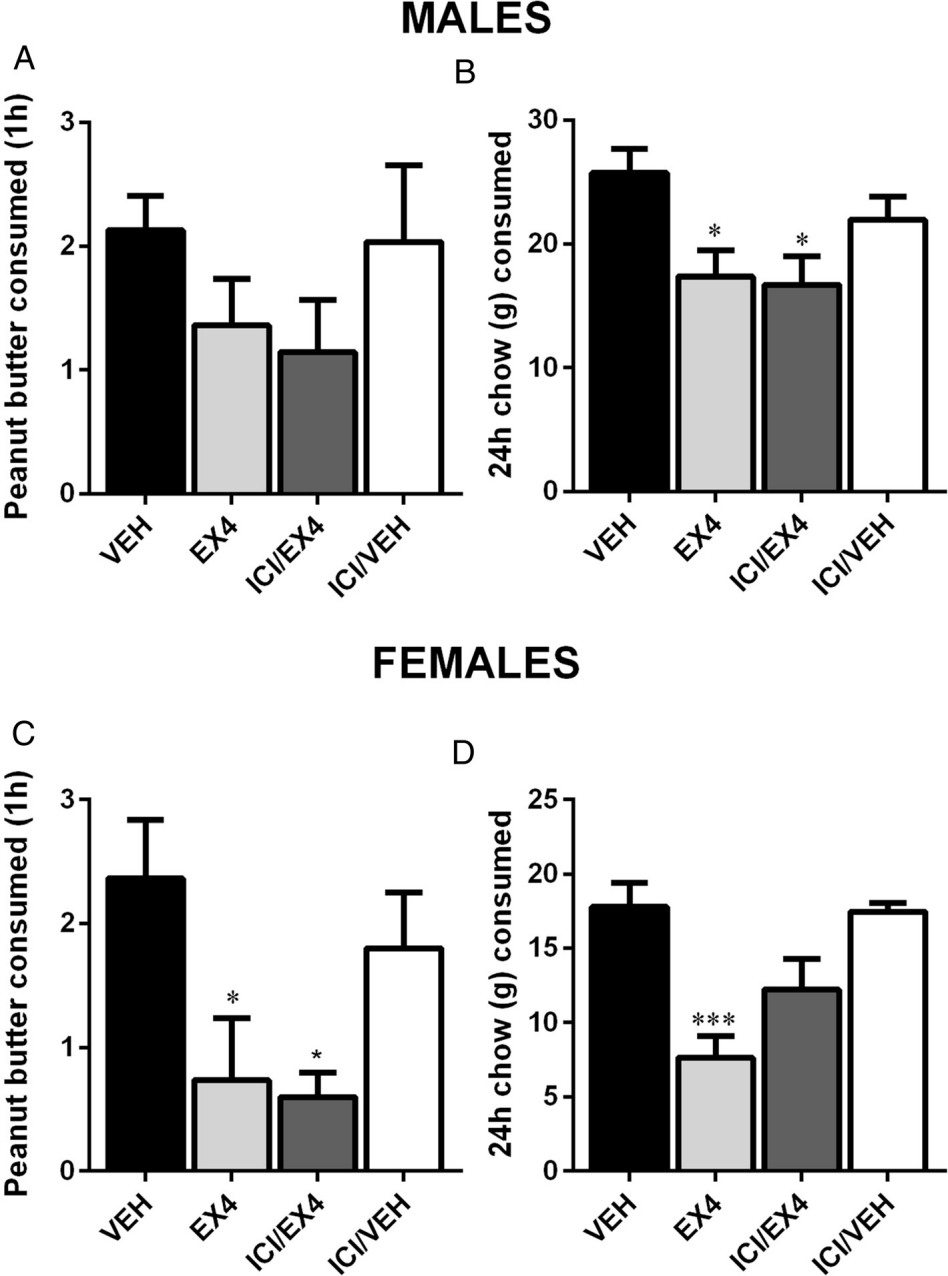
Effect of central estrogen blockade on the actions of Ex4 on peanut butter consumption or chow intake is not divergent between the sexes. No significant changes in peanut butter consumption were observed in males (**a**), but a significant reduction in consumption was noted in females after injection of Ex4 or Ex4 and ICI (**c**). Ex4 reduced chow intake in both males and females (**b**, **d**). This reduction was attenuated by simultaneous ICI treatment only in females. Chow and peanut butter consumption are displayed in grams. Data are expressed as mean ± SEM. *n* = 10 (females) and 9 (males) per treatment group for peanut butter measurement and *n* = 10 (females) and 18 (males) per treatment group for chow intake. *p < 0.05, ***p < 0.001

Two-way repeated measures ANOVA indicated that the main effect of sex (*F*_(1, 63)_ = 0.8913, *p* > 0.1) was not significant. Drug treatment (*F*_(3, 63)_ = 4.682, *p* < 0.01) was significant. A significant interaction between these two factors was not found (*F*_(3, 63)_ = 0.3939, *p* > 0.1). Treatment with Ex4 alone, or in combination with the estrogen antagonist ICI, led to a significant reduction in peanut butter consumption after 1 h in females (one-way ANOVA, *F*_(3, 33)_ = 4.242, *p* < 0.05; Fig. 4c), but not in males (*F*_(3, 30)_ = 1.142, *p* > 0.1; Fig. 4a). Chow consumption was significantly different, both regarding the differences in the effect of sex (*F*_(1, 97)_ = 17.73, *p* < 0.0001) and the main effect of drug treatment (*F*_(3, 97)_ = 1.197, *p* < 0.001). A significant interaction between these two factors was not found (*F*_(3, 97)_ = 0.6991, *p* > 0.1). Twenty-four hour chow consumption was reduced after Ex4 treatment in both male and female subjects (one-way ANOVA, *F*_(3, 66)_ = 4.139, *p* < 0.05; Fig. 4b, *F*_(3, 31)_ = 9.230, *p* < 0.001; Fig. 4d, for males and females, respectively). In contrast to peanut butter consumption, ICI treatment attenuated Ex4-induced food intake reduction in females, but not in males, resulting in an increased amount of chow intake consumed during the 24-h period (Fig. 4b, d).

### Effects of central administration of the ERα-antagonist MPPd on male and female food-motivated behavior after Ex4 treatment

**Fig. 5:**
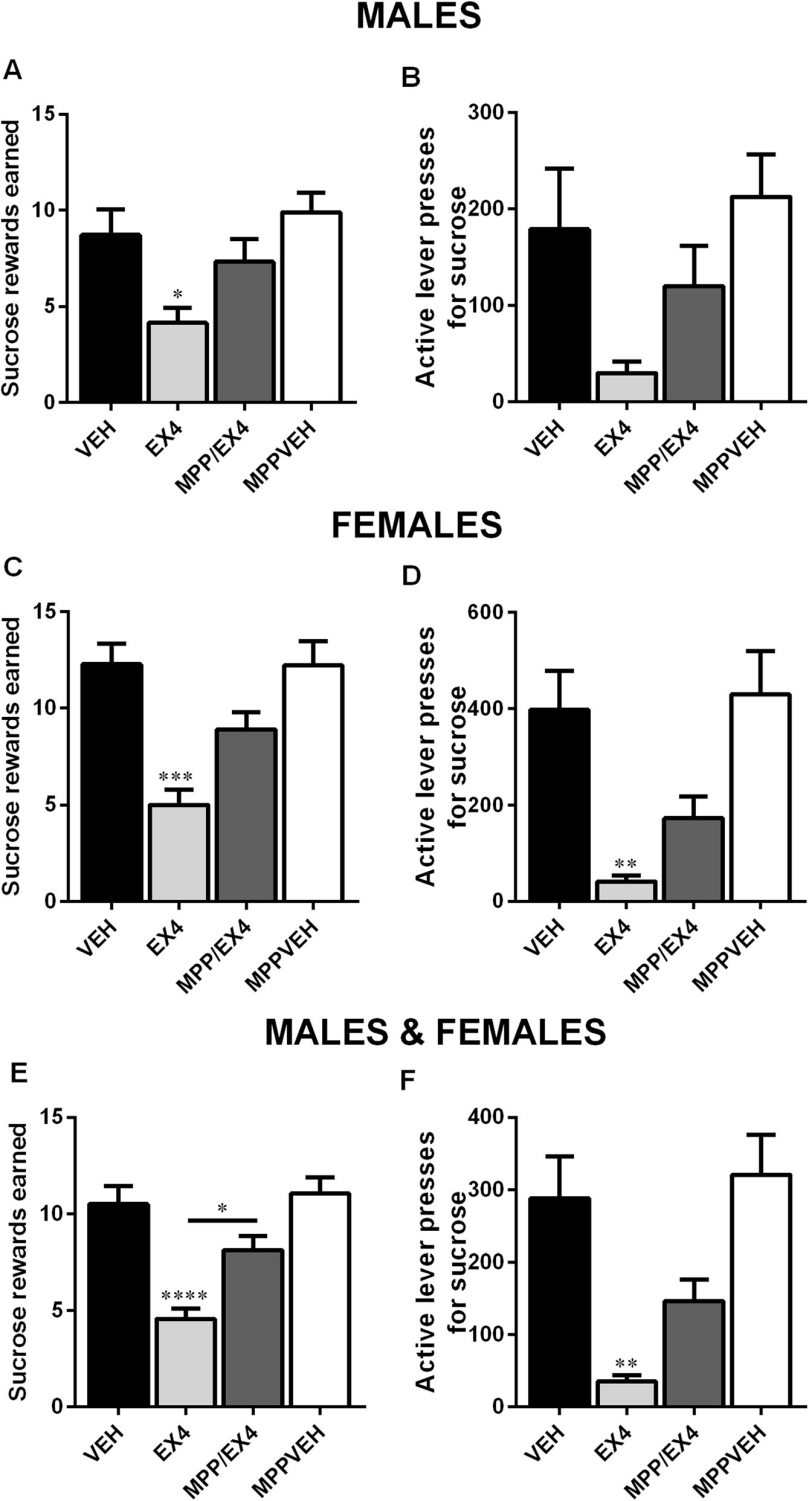
Central injection of the ERα-antagonist MPPd attenuated the effects of Ex4 on food-motivated behavior. As expected, central administration of Ex4 led to a reduction in sucrose rewards earned in females (**c**) and males (**a**). Ex4 no longer significantly reduced sucrose rewards earned after administration of the specific ERα-antagonist MPPd. Additionally, Ex4 treatment reduced lever presses for sucrose earned in females (**d**) and produced a trend for reduction in males (**b**), an effect which was also attenuated by co-treatment with MPPd. Compilation of female and male data revealed a significant reduction in sucrose rewards earned (**e**) and active lever presses (**f**) after EX4 treatment that was attenuated after combined MPP/Ex4 treatment. Data are expressed as mean ± SEM. *n* = 7–9 for males and females. *p < 0.05, **p < 0.01, ***p < 0.001, ****p < 0.0001

For sucrose rewards earned, two-way ANOVA revealed a significant difference in the main effect of sex (Holm-Sidak’s multiple comparisons test, *F*_(1, 56)_ = 7.319, *p* < 0.01) and effect of drug treatment (*F*_(3, 56)_ = 13.98, *p* < 0.0001). A significant interaction between these two factors was not found (*F*_(3, 56)_ = 0.5209, *p* > 0.1). As shown previously, Ex4 significantly decreased the amount of sucrose rewards earned in both male and female rodents in the operant conditioning task (one-way ANOVA, *F*_(3, 28)_ = 4.750, *p* < 0.5; Fig. 5a, *F*_(3, 28)_ = 10.04, *p* < 0.001; Fig. 5c, for males and females, respectively). Coinciding treatment with MPPd attenuated the effects of Ex4 in both sexes (Fig. 5a, c). Two-way repeated measures ANOVA revealed that the main effect of sex (*F*_(1, 56)_ = 9.483, *p* < 0.01) and drug treatment (*F*_(3, 56)_ = 10.36, *p* < 0.0001) were significant for active lever presses. There was, however, no interaction between these two factors (*F*_(3, 56)_ = 1.777, *p* > 0.1). Active lever presses for sucrose were significantly decreased in females after Ex4 treatment (one-way ANOVA, *F*_(3, 28)_ = 7.328, *p* < 0.01; Fig. 5d), but not in males (Fig. 5b). The effects of Ex4 on lever presses were also attenuated after MPPd treatment in females and an increase in presses was also present in males, though this increase was not significant (Fig. 5d, b). Combined results for males and females showed a significant increase in sucrose rewards earned after MPPd/Ex4 treatment compared to Ex4 treatment alone (one-way ANOVA, *F*_(3, 60)_ = 12.92, *p* < 0.05; Fig. 5e). The attenuation of MPPd/Ex4 administration on active lever presses in males and females combined, however, did not reach significance (5F).

**Fig. 6:**
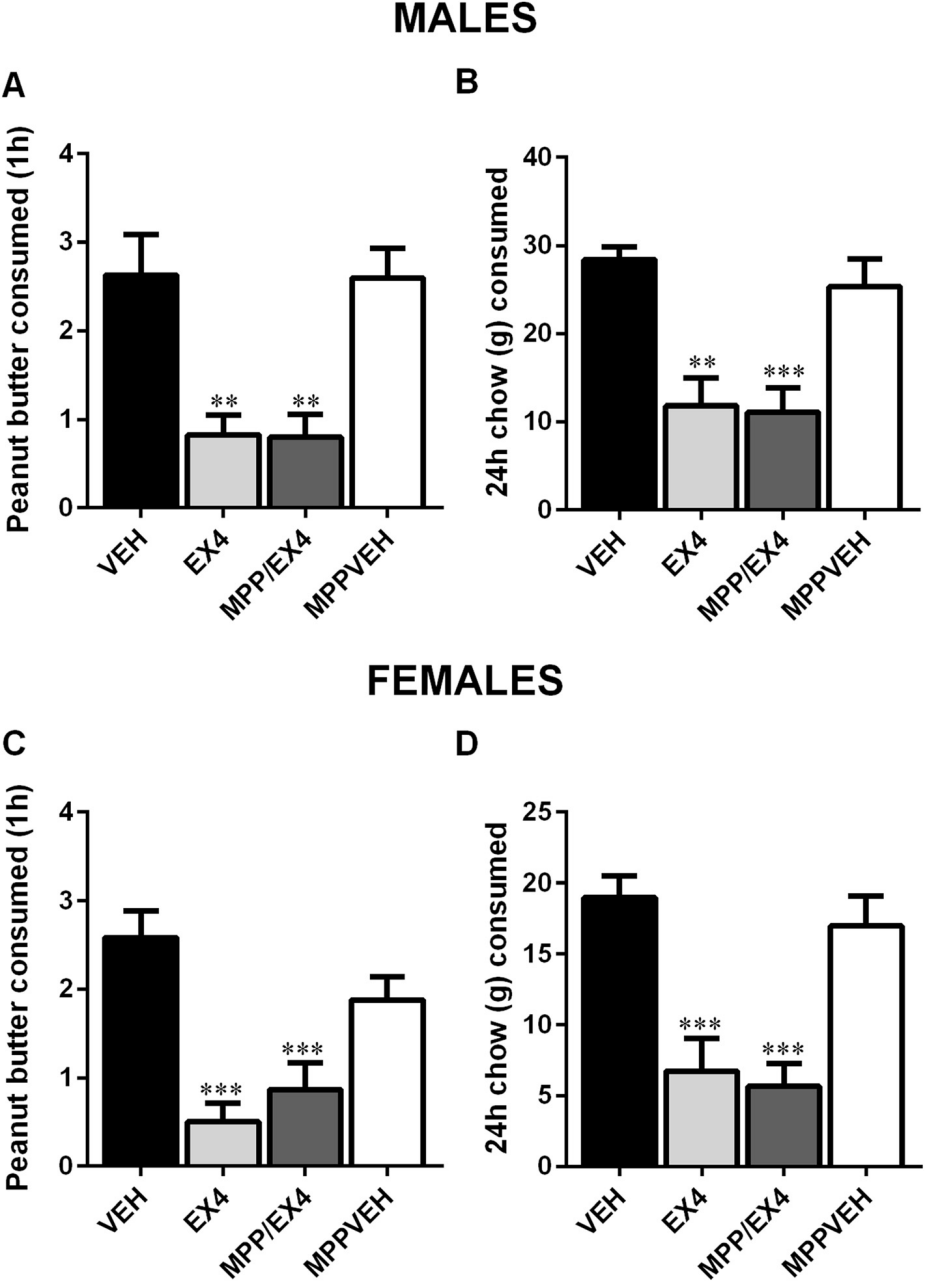
MPPd treatment did not attenuate the effects of central Ex4 treatment on food intake. Central injection with the GLP-1R agonist Ex4 significantly reduced peanut butter intake and chow intake in males (**a**, **b**) and females (**c**, **d**). The reduction was still present after treatment with the ERα-antagonist MPPd. Chow and peanut butter consumption are displayed in grams. Data are expressed as mean ± SEM. *n* = 7–9 for males and females. **p < 0.01, ***p < 0.001

Two-way repeated measures ANOVA indicated that the main effect of sex was not significant (*F*_(1, 56)_ = 1.413, *p* > 0.1) for 1-h peanut butter consumption. The main effect of drug treatment was significant (*F*_(3, 56)_ = 20.13, *p* < 0.0001). A significant interaction between these two factors was not found (*F*_(3, 56)_ = 0.7364, *p* > 0.1). MPPd injection did not affect the action of Ex4 on peanut butter consumption which remained significantly reduced compared to vehicle in both males and females (one-way ANOVA, *F*_(3, 28)_ = 10.21, *p* < 0.01; Fig. 6a, *F*_(3, 28)_ = 10.74, *p* < 0.001; Fig. 6c, for males and females, respectively). Two-way repeated measures ANOVA revealed that the main effect of sex (*F*_(1, 56)_ = 16.94, *p* < 0.001) and drug treatment (*F*_(3, 56)_ = 21.27, *p* < 0.0001) were both significant for the 24-h food consumption. There was no interaction between these two factors (*F*_(3, 56)_ = 0.3711, *p* > 0.1). Furthermore, the 24-h food intake was also unaffected after combined Ex4 and MPPd treatment compared to Ex4 alone and was still significantly reduced compared to controls (one-way ANOVA, *F*_(3, 28)_ = 9.905, *p* < 0.001; Fig. 6b and *F*_(3, 28)_ = 12.83, *p* < 0.0001; Fig. 6d, for males and females, respectively). Two male and two female rats did not finish the study due to dislodged or blocked cannula.

## Discussion

Preclinical research unraveling the neural basis of feeding behavior is almost exclusively conducted in male subjects, although recent evidence suggests females and males may regulate feeding behavior differently [4–6]. Likewise, reward-mediated behavior may be differentially regulated between men and women due to the impact of sex hormones [40, 41]. GLP-1 agonists have previously been shown to affect food intake and food-reward behavior [27, 29, 42, 43]. The current study provides evidence that Ex4, the long-lasting GLP-1 analog, may have differential effects on food-reward in male and female subjects. Moreover, we demonstrate that signaling via central ER is necessary for the full impact of Ex4 on food reward. More specifically, signaling at the ERα may be crucial, since selective ERα-antagonist, MPPd, attenuated Ex4-induced food reward suppression.

Central GLP-1R agonist injection potently reduced food-motivated behavior in females and males in a sex-dependent manner in a progressive ratio operant conditioning task. The progressive ratio operant conditioning task, used here to analyze reward behavior, measures the motivation to obtain a food reward. Food-reward behavior is typically divided into two components: liking and wanting, where liking is typically associated with the immediate experience the moment a palatable food is consumed, while wanting is associated with reward seeking and an increased motivation to obtain rewarding foods [23]. Here, we show that the wanting component of the food-reward suppressing effects of Ex4 displays differential sensitivity in male and female rats. The actions of Ex4 on food reward were also accompanied by a reduction in palatable food intake in females and males compared to controls. However, peanut butter consumption response to Ex4 was not significantly different between male and female subjects. Progressive ratio test and palatable food intake may to some extent represent the two different aspects of food reward, where operant conditioning mainly measures wanting, while peanut butter intake better reflects the liking aspect of food reward. The differences in sensitivity to Ex4 treatment between sexes, therefore, seem to be specific to food-motivated behavior. Though, it is important to note that consumption of peanut butter is not a “pure” test of liking as it allows for ingesting ad libitum amounts of the palatable food; thus, an interaction with satiation signaling cannot be discounted.

Our data clearly indicate differences in the sensitivity to the food reward impact of Ex4 between females and males. Interestingly, only females showed a significant reduction in active lever presses, and sucrose rewards earned, in the non-food deprived state after Ex4 treatment. Yet, Ex4 has previously been shown to reduce food-motivated behavior and reduce the intake of palatable foods in males [27, 44]. However, considering that the preceding studies were conducted in rats that were food-restricted during training, and typically also testing in the progressive ratio task, in contrast to this experiment, the absence of reward reduction in males may indicate that Ex4 effect on food reward may be dependent on feeding state. A recent report indicates that a single overnight fast can attenuate GLP-1’s suppressive effect on food intake. [45]. Here, we show similar results for the GLP-1 agonist, Ex4, as unrestricted access to chow resulted in a more potent reduction in chow intake after GLP-1R agonist treatment. For reward behavior, the interaction with fasting differs: overnight food restriction led to a significant reduction in sucrose rewards earned in male subjects after Ex4 administration. This effect was still less potent than in females, further indicating a higher sensitivity to Ex4 in female subjects. Baseline measures for reward slightly differed between sexes, with females receiving a larger quantity of rewards after vehicle treatment. The differential baseline values may cause some difficulty when comparing the effects of Ex4 in each sex since the higher baseline values displayed in females may make small reductions in reward caused by the drug more apparent. However, Ex4 treatment still reduced sucrose rewards earned in the food-restricted females to a larger extent than in food-restricted males, even though here the baseline values of rewards earned are not significantly different between the sexes. This further supports the hypothesis that females are more greatly affected by Ex4 treatment than their male counterparts.

Sex differences in reward were not accompanied by differences in overall food intake, indicating a potential selectivity of the sex differences to reward. The clear impact of Ex4 treatment on food intake suggests that the drug was in fact also effective in males, as expected based on previous studies with this agonist. However, the long-term interaction of estrogens with GLP-1 in women, including potential resulting sex differences in body weight, requires further investigation. Our results clearly suggest that when food availability is limited by the amount of work/effort the animal has to put in to obtain the food, Ex4 is much more efficient at reducing this type of behavior in females. Since food is rarely freely available to animals in nature, this effort-based eating behavior is likely very relevant to natural eating behavior. Surprisingly, only a few studies have looked at potential differences in food reward regulation between sexes in humans, though a recent study revealed differential connectivity in areas of the brain regulating reward between men and women which may indicate that food-reward behavior is differently regulated between sexes [46]. Recently, several studies investigated the effects of GLP-1R agonists on food intake and reward, and activation of intake or reward-controlling CNS areas in humans [47–49]; however, sex or gender interaction was not the focus, and therefore, sex was either not reported or the effect of sex not analyzed.

While the ability of estrogens to increase the potency of Ex4 in both sexes is unexpected, it is in line with one previous study indicating that co-administration of a GLP-1 and estrogen agonist in a conjugated form, that activates cells expressing estrogen receptors and GLP-1R, or GLP-1R alone, reduces food intake and body weight at doses of the hormones that are ineffective alone [50]. Importantly, the reduction was comparable in both sexes, suggesting that females and males benefit from the enhancing effects of estrogens on GLP-1 action. These data combined with current findings suggest that estrogens’ signaling is sufficient to enhance food intake in both sexes but does not seem necessary for the anorexic effect of Ex4.

Estrogen receptors and GLP-1R are co-localized in areas involved in reward behavior regulation such as the VTA and the NAc [26, 51]. To our knowledge, little is known about the role of estrogens in food reward, though estradiol treatment in ovariectomized females was previously shown to reduce the intake of palatable, high-fat/high-sugar foods, in addition to reducing intake of standard food [52]. The role of GLP-1R activation in reward, however, is well-supported by recent literature [27, 29, 44], though studies investigating the food-reward-mediated effects of GLP-1 have previously been conducted exclusively in males [27, 31, 53, 54]. Nonetheless, estrogens have been shown to cause functional changes in GABAergic neurons in the VTA and dopaminergic terminals within the striatum [55, 56], and estrogen treatment enhances striatal dopamine release [55, 57–61]. Furthermore, estrogens induce increased dopaminergic sensitivity to cocaine in ovariectomized rats [61]. In addition, administration of estradiol during the follicular phase increases the subjective effects of amphetamine [62]. Levels of dopamine, its metabolites and synthesizing enzymes, and amphetamine-induced dopamine release vary as a function of the ovarian cycle stage [57, 63]. Women progress faster to cocaine dependence and display a higher incidence of relapse; female rats also display a higher preference for cocaine rewards, potentially due to the effects of estrogens [40, 41]. In contrast, gonadectomy and sex hormone treatments in males do not have a large impact on cocaine-reinforced behaviors [64, 65]. Thus, while the food reward impact of estrogens is unknown, the literature at least suggests its impact on drug reward. Past studies focus on the rewarding effects of drugs of abuse where estrogens mainly enhance reward in contrast to the current results where estrogens reduced food reward by enhancing effects exerted by GLP-1. Interestingly, it has previously been reported that women report higher levels of craving to rewarding foods, such as chocolate; these cravings were reported to be most persistent during the periods of the menstrual cycle characterized by low circulating levels of estrogens [66] indicating that estrogens may indeed reduce food reward, in contrast to their effects on drug reward.

Current experiments utilizing ICI, a nonselective ER antagonist, revealed that estrogens may increase the sensitivity to Ex4 effects on reward. Estrogens’ effects on food intake may be due to its ability to modulate the activity of endogenous hormones [4, 5]. Thus, surprisingly, the impact of estrogens on the anorexic action in the case of insulin is opposite to that previously seen for leptin and currently revealed for Ex4. Collectively, these studies illustrate estrogen’s ability to modulate feeding behavior by acting on appetite-regulating hormones. Therefore, we hypothesized that estrogens may also play a role in the differential sensitivity displayed by males and females after Ex4 treatment. As hypothesized, central treatment with the ERα (Esr1) and ERβ (Esr2) receptor antagonist, ICI, attenuated the food reward effects of Ex4 in females, but surprisingly, food-reward behavior in males was also attenuated by ER blockade. Past literature suggests that ERα is involved in homeostatic feeding and weight regulation, and deletion of ERα leads to obesity in both sexes in mice [67, 68]. In addition, administration of estradiol reduces food intake and prevents weight gain in wild-type, but not in ovariectomized transgenic mice lacking ERα, further indicating a role for ERα in homeostatic feeding [69, 70]. Previous studies have mostly failed to find a connection between ERβ and feeding behavior [69, 70]. In contrast, selective activation of ERβ receptor was even shown to increase cocaine-seeking behavior, an effect that was not seen after ERα receptor stimulation, indicating that ERβ may affect reward-related behavior [71]. Thus, ERβ was an unlikely interaction target. Consistent with this idea, the selective ERα-antagonist MPPd was sufficient to attenuate the effects of Ex4 on reward, in both males and females, indicating that signaling via the ERα may be necessary for Ex4 impact on food-reward behavior. Though it is important to note that while MPPd is classified as an ERα antagonist and has been shown to antagonize estrogen-regulated genes in vitro and attenuate estrous-related decreases in food intake [72, 73], it has also been suggested to have estrogen-like activity since it increased uterine weight in rodents and reduced food intake in OVX rats [73]. Our data do not eliminate the possible involvement of ERβ, or other ER, such as GPR30. It is also possible that other gonadal steroids may be behind these differences. Testosterone, for example, regulates insulin sensitivity [74, 75]. However, in contrast to ovariectomy, orchiectomy decreases daily food intake and body weight by decreasing meal frequency, an effect which can be reversed by testosterone treatment [76, 77]. Potential effects of testosterone on Ex4 actions on food reward are yet to be investigated. Importantly, substances other than sex steroids may also be involved.

Although females have higher estrogen levels than males, males also possess a certain level of circulating estrogens due to the conversion of testosterone to estrogen, by the enzyme aromatase, in the reproductive tract, bone, and adipose tissue [78]. Since premenopausal females have much higher levels of circulating estrogens, the increased impact of Ex4 in females shown here may result from these elevated levels of estrogens. However, since estrogens can enhance the action of GLP-1 on food intake in males [50] and blockade of ER signaling reduces the impact of GLP-1R activation on food reward in males, even low levels of estrogens in males may be sufficient to increase the sensitivity to GLP-1. Moreover, the estrogen synthesis enzyme aromatase is also present in the brain, mainly in neurons in the hypothalamus and limbic system [79]. In addition, the anorexigenic effects of estradiol have previously been attributed to its actions within the CNS as only central but not peripheral blockade with ICI increases food intake [38]. Thus, it is possible that the small amounts of estrogens synthesized by the male brain are also sufficient to play an important role in the reward effects of GLP-1. It is even possible that brain-synthesized estrogens may function as a potential downstream mediator of GLP-1 effects, an idea ripe for future studies.

The current study provides novel evidence for the effects of the synthetic GLP-1R agonist Ex4 on food-motivated behavior in females and its interaction with central estrogen signaling, but it also has some limitations. Here, for reasons described above, only central Ex4 application was used in contrast to the peripheral administration utilized during clinical treatment with these agonists. However, both GLP-1 and Ex4 have been shown to cross the blood-brain barrier [35, 80], and feeding or reward impact of Ex4 is mediated by CNS GLP-1R, indicating that GLP-1 agonists are likely to result in sex differences even after peripheral injection. In addition, the potential food-reward suppressing effects of endogenously produced GLP-1 may differ from those found here for Ex4; thus, the results here are more directly relevant to the clinically used GLP-1 analog, and the relevance to endogenous GLP-1 should be a topic for future investigation.

## Conclusions

These data indicate that there are sex differences in the sensitivity to the food reward effects of Ex4. Moreover, signaling through the ERα is essential for the reward-reducing impact of Ex4. Synthetic GLP-1 analogs are widely used in the clinic as a treatment for type-2 diabetes, and one was also recently approved as a supplement for weight-management [81]. Although these drugs are prescribed to individuals of both sexes, preclinical studies have almost exclusively been done in male subjects, a concern which is present throughout many scientific fields. Moreover, these drugs have the ability to cross the blood-brain barrier and exert the effects evaluated here through their actions on the CNS. Data presented here may indicate a higher sensitivity to these substances in women. If our findings will be replicated in a future clinical study, then physicians prescribing this medication may need to consider establishing alternative doses for female patients taking these drugs.
